# Clinically diagnosed primary transitional cell carcinoma of the colon: A case report

**DOI:** 10.1016/j.ijscr.2019.01.035

**Published:** 2019-01-31

**Authors:** Ryuichi Taketomi, Naoto Ujiie, Yoshitaka Enomoto, Naruhito Takido, Yasushi Kawaharada, Masashi Zuguchi, Yosuke Kubota, Ken Saito

**Affiliations:** Division of Surgery, Hiraka General Hospital, Yokote, Akita, Japan

**Keywords:** Transitional cell carcinoma, Colon cancer, Primary

## Abstract

**Introduction:**

Most transitional cell carcinomas (TCCs) occur in the urinary tract. There are no reports of TCC originating in the colon. This report presents a very rare case of TCC that primarily occurred in the colon.

**Presentation of case:**

A 78-year-old female presented with adenocarcinoma of the rectum and TCC of the ascending colon. She was screened for urologic and gynecologic carcinomas because the TCC was considered a metastatic lesion; however, cytodiagnosis of urine, the cervix and corpus uteri revealed no abnormal findings. An operation was performed, and histological examination revealed adenocarcinoma of the rectum and TCC of the ascending colon. Immunohistochemical stained specimens of the ascending colon revealed tumor cells of cytokeratin (CK) 7−/CK20+ pattern. Eleven months post-operation, a metastatic TCC was found in the liver. The patient was treated with chemotherapy; however, she died 19 months after the operation.

**Discussion:**

Our case was clinically considered that the TCC primarily occurred in the colon after analyzing the results of several examinations. Immunohistochemical staining of CK7 and CK20 expression pattern also suggested that the TCC of the ascending colon originated in the colon.

**Conclusion:**

To the best of our knowledge, this is the first literature report of TCC that originated in the colon. TCC that primarily occurs in the colon may rapidly progress, as in the case presented. Therefore, it is necessary to establish more appropriate treatment for similar cases.

## Highlights

• This is the first literature report of TCC that originated in the colon.
• Our case was clinically considered that the TCC primarily occurred in the colon.
• CK7 and CK20 expression pattern also suggested that the TCC originated in the colon.

## 1 Introduction

Transitional cell carcinoma (TCC) is an epithelial malignant tumor derived from transitional epithelial tissue, and 90% of TCCs are localized in the bladder [1]. Some reports describe that TCCs primarily occur in the ovaries [2,3]. However, to the best of our knowledge, there are no reports of TCC primarily occurring in the colon. Our study demonstrates a novel presentation of TCC that is considered to have originated in the colon. This work has been reported in line with the SCARE criteria [4].

## 2 Presentation of case

**Fig. 1:**
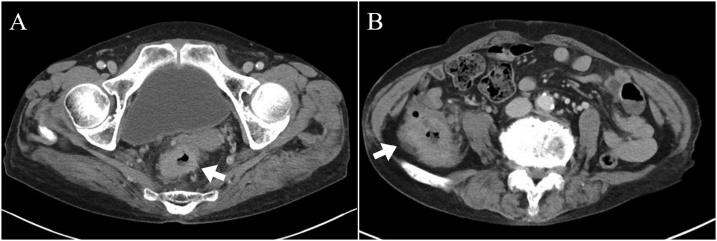
Computed tomography (CT). CT shows a mass in the rectum (A) and ascending colon (B).

A 78-year-old female presented to our hospital because her laboratory test data showed anemia and fecal occult blood test was positive. Lower digestive tract endoscopy showed a circumferential tumor in the rectum at 5 cm from the anal verge and a type 3 tumor of the ascending colon at 2 cm from the ileocecal valve. Endoscopic biopsy diagnosed the rectal and ascending colonic lesions as adenocarcinoma and TCC, respectively. Elevation in the levels of tumor markers such as carcinoembryonic antigen (CEA), carbohydrate antigen (CA) 19-9, and CA 125 was not observed. Computed tomography (CT) revealed masses in the rectum and ascending colon as well as several regional lymph node enlargements at each lesion (Fig. 1). The patient was clinically diagnosed with a T4aN1bM0, stage IIIB adenocarcinoma of the rectum and a T4aN2aM0, stage IIIC TCC of the ascending colon [Union for International Cancer Control, 8th version] [5].

We suspected that the ascending colonic lesion was a metastatic urologic or gynecologic carcinoma; therefore we performed screening. Cytodiagnosis of urine revealed Class II cells, and cystoscopy revealed no abnormal findings. Transvaginal ultrasonography also exhibited no abnormal findings. Cytodiagnosis of the cervix and corpus uteri revealed Class II cells, respectively. Positron emission tomography-CT (PET-CT) did not detect any abnormal findings except colonic lesion. Therefore, the ascending colonic lesion was clinically considered as a lesion of primary TCC. Subsequently, Hartmann’s operation and ileocecal resection with radical lymph node dissection were performed. Both lesions were resected getting enough distance from the tumor according to JSCCR guidelines 2016; oral/anal margin was 10/2 cm in the rectal lesion and 10/10 cm in the ascending lesion [6].

**Fig. 2:**
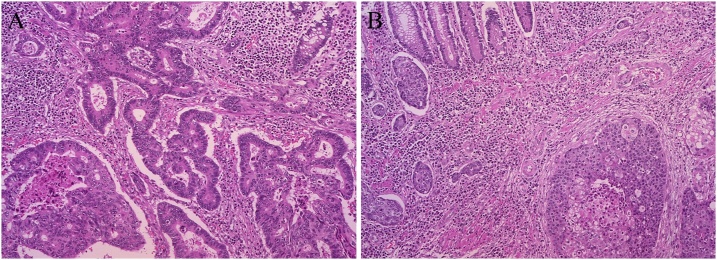
Photomicrographs of rectal (A) and ascending colonic (B) histology (Elastica-Masson staining, 100×). (A) shows a well-differentiated tubular adenocarcinoma, but (B) shows growth of transitional cells in the mucosa.

**Fig. 3:**
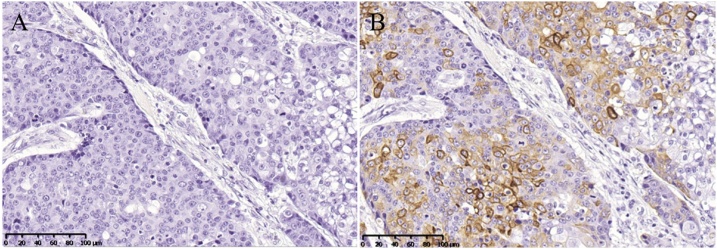
Immunohistochemical studies of ascending colonic histology [A: CK 7 (400×); B: CK 20 (400×)]. Tumor cells were negative for CK 7 (A) and positive for CK 20 (B).

Histological examination revealed adenocarcinoma of the rectum and TCC of the ascending colon (Fig. 2). Oral and anal resection margins of the rectal and ascending colonic legion were both negative. Nine lymph node metastases in the proximal mesentery of the rectum or along the superior rectal artery and three lymph node metastases in the proximal mesentery of the ascending colon or along the ileocecal artery were detected. The definitive diagnosis of the rectal lesion was pT3N1bM0, stage IIIB, while that of the ascending colonic lesion was pT3N2bM0, stage IIIC. Immunohistochemically stained specimens of tumor cells of the ascending colonic lesion were negative for cytokeratin (CK) 7 but positive for CK 20 (Fig. 3). No postoperative complication was observed, and diet was started on postoperative day 4. After acquiring self-care for colostomy, the patient was discharged on postoperative day 22.

Eleven months post-operation, space-occupying lesion in S5 of the liver was detected by CT. Percutaneous biopsy of the hepatic lesion indicated metastasis of the TCC. The patient was intravenously treated with chemotherapy as follow regimen: gemcitabine 1000 mg/m^2^ (on days 1 and 8) and carboplatin area under the curve = 5 (on day 1) according to treatment protocols for bladder cancer [7]. Two months after receiving chemotherapy, the size of the hepatic tumor increased and her general condition deteriorated. The patient died 19 months after the operation.

## 3 Discussion

TCCs can occur anywhere along the urinary tract, among which 90% are localized in the bladder, and approximately 90% of colorectal cancers are adenocarcinomas [1,8]. In the present case, TCC of the ascending colon was initially considered to be a metastatic lesion from a urologic or gynecologic carcinoma. Cytodiagnosis of urine, cystoscopy, transvaginal ultrasonography, cytodiagnosis of the cervix and corpus uteri were performed; however, obvious malignancy was not observed. Moreover, PET-CT did not show any abnormal findings except colonic lesion. On the basis of these test results, TCC of the ascending colon was clinically dismissed as metastasis from other organs, and we considered that TCC originated in the colon.

Pathological findings revealed by immunohistochemical staining of resected specimens of the ascending colon also suggested that TCC originated in the colon. Immunohistochemical staining of CK is useful for making the diagnosis of epithelial occult primary cancer [9]. The immunohistochemical staining that combines CK7 and CK20 is widely used. Their expression patterns help to distinguish the site of origin of metastatic carcinomas [10]. Most primary colorectal carcinomas show a CK7−/CK20+ pattern, and most primary urothelial carcinomas show a CK7+/CK20+ pattern [10,11]. In our case, on staining specimens of the ascending colon lesion, the tumor cells were CK7−/CK20+. These results suggested that TCC of the ascending colon originated in the colon. In ovarian cancer, TCC represents a poorly differentiated form of high-grade serous carcinoma [12]. Primary peritoneal carcinoma presents high-grade serous carcinoma [13]. It might be a possibility, therefore, that primary peritoneal carcinoma invaded the colon and presented TCC in the colon.

In the present case, because liver metastasis was TCC, chemotherapy was administered according to treatment protocols for bladder cancer [7]. The patient survived for 2 months after chemotherapy. It is an undeniable possibility that survival may have been prolonged if chemotherapy was performed according to the treatment guidelines for colon cancer. Therefore, it is necessary to establish more appropriate treatment protocols for similar cases.

## 4 Conclusion

This case presents a rare incidence of TCC that is considered to have originated in the colon. It is an undeniable possibility that TCC primarily occurring in the colon progresses rapidly, as was evident in our case. Therefore, adequate therapeutic strategy should be established for similar cases.

## Conflicts of interest

All authors have no conflicts of interest to declare.

## Sources of funding

This research did not receive any specific grant from funding agencies.

## Ethical approval

Ethical approval has been exempted from our institution for this case report.

## Consent

Written informed consent was obtained from the patient’s family for the publication of this case report and accompanying images.

## Author’s contribution

RT, NU, and YE reviewed the patient and discussed the literature review. RT, YE, NT, YK, MZ, YK, and KS treated the patient (operation and chemotherapy). RT and NU wrote the manuscript draft. All authors reviewed and edited the manuscript.

## Registration of research studies

This paper is a clinical report, no research involved.

## Guarantor

Naoto Ujiie and Yoshitaka Enomoto.

## Provenance and peer review

Not commissioned, externally peer-reviewed.
